# Incidence of Emergency Department Visits for Electric Rental Scooters Using Detailed Ridership Data

**DOI:** 10.5811/westjem.2021.6.51101

**Published:** 2022-01-31

**Authors:** Chelsea Williams, Cindy C. Bitter, Steven Lorber, Caleb R. Overfelt, Holly Zehfus, Andrea Spangler, Valerie Lew, Lawrence M. Lewis, Rosanne S. Naunheim

**Affiliations:** * University of Washington, Department of Emergency Medicine, Seattle, Washington; † Saint Louis University School of Medicine, Division of Emergency Medicine, St. Louis, Missouri; ‡ Washington University in St. Louis/Barnes Jewish Hospital, Department of Emergency Medicine, St. Louis, Missouri

## Abstract

**Introduction:**

Electric scooter (e-scooter) rental usage has increased exponentially around the country, expanding to more than 120 cities by the end of 2018. Early attempts to capture the safety effects of widespread adoption of this technology have been hampered by lack of accurate ridership data. Here we describe a 17-month evolution of ridership characteristics in St. Louis, Missouri, and the frequency of e-scooter rental-related injuries serious enough to require an emergency department (ED) visit over this time frame; we also provide estimates of incidence rates of injuries based on company ridership data.

**Methods:**

We performed a combination retrospective chart review and prospective questionnaire-based analysis of adult e-scooter rental-related ED visits in both downtown St. Louis Level 1 trauma centers during the first 17 months of e-scooter rental usage (August 2018–December 2019). The retrospective portion focused on demographics, alcohol use, helmet use, disposition, operative repair, and temporal and severity markers. The prospective portion focused on more detailed crash and rider data. Finally, we used ridership data from both e-scooter rental companies in St. Louis to estimate incidence and temporal trends.

**Results:**

A total of 221 patients had e-scooter rental-related ED visits. The median age of our population was 31 years with 58.8% male and 53.8% White. There were no deaths. Ninety-two patients were found to have fractures with 38% requiring surgery. Of the 21 patients diagnosed with head injury, five had an intracranial bleed. Overall incidence of ED visits related to e-scooters was 2.1 per 10,000 trips and 2.2 per 10,000 miles with the number of ED visits by month closely correlated with the number of rides per month (Pearson correlation coefficient = 0.95).

**Conclusion:**

The number of e-scooter rental-related injuries seen in St. Louis trauma centers was relatively low and correlated closely with overall number of rides. The number of injuries decreased and were less severe from 2018 to 2019 with infrequent intracranial injuries and a large percentage of fractures requiring operative repair.

## INTRODUCTION

The first standing electric scooter (e-scooter) rentals were introduced in the United States (US) in September 2017 and expanded to more than 120 cities by the end of 2018.^1^ The first death in the US was reported in 2018, and there have been several case series of injury patterns related to their use.^2^,^3^,^4^,^5^,^6^,^7^ Two prior nationwide studies used queries of the National Electronic Injury Surveillance System (NEISS) to estimate injury rates from e-scooters, but the time frame included only the first five months of e-scooter rentals.^8^,^9^ These early attempts to capture the effects of widespread adoption did not fully capture the period of rapid growth of e-scooter rentals. However, a subsequent query of NEISS showed a sevenfold increase in injuries related to e-scooters between 2014–2019, before and after e-scooter rentals became widespread.^8^,^10^ No studies have compared incidence of injuries or ridership data in the same locale over an extended period of time. We are the first to report detailed e-scooter rental-related injury incidence rates based on company ridership data.

We followed e-scooter rental-related injuries at the two downtown St. Louis emergency departments (ED) since Lime (Neutron Holdings, Inc., San Francisco, CA) and Bird (Bird Rides, Inc., Santa Monica, CA) e-scooter rentals first arrived in our metropolitan area in August 2018. We describe the 17-month evolution of ridership characteristics in the city, the frequency of e-scooter rental-related injuries serious enough to require an ED visit over this time frame, and provide estimates of the incidence rate of injuries based on company ridership data.

## METHODS

We performed a retrospective analysis of all patients 18 years or older presenting to the Barnes Jewish Hospital (BJH) or the Saint Louis University Hospital (SLUH) EDs for an e-scooter related injury during the first five months (August–December 2018) of e-scooter rental rollout in the St. Louis metropolitan area. These facilities are the two major adult hospitals and only Level 1 trauma centers located within the city limits. We then performed a prospective analysis of patients meeting the same selection criteria for calendar year 2019 at BJH and continued the retrospective review at SLUH through calendar year 2019. The study had institutional board review approval from Washington University in St. Louis and from Saint Louis University with waiver of consent for SLUH given the retrospective nature of the study. Our methods and results are reported as per the guidelines of the STROBE (Strengthening the Reporting of Observational Studies in Epidemiology) checklist.^11^

**Figure 1:**
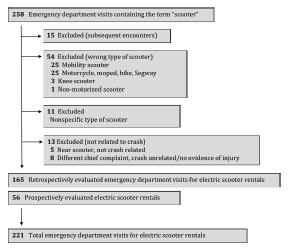
Identifying total emergency department visits for electric scooter-related injury.

For the retrospective component of the study, we queried the electronic health record for all ED visits that contained the word “scooter” in the diagnosis, chief complaint, or clinical notes. Charts were included for review if documentation noted any of the following keywords: “electric,” “rental,” “standing motorized,” “lime” or “bird.” Lime and Bird were the only two companies offering rentals during the study period and thus the terms were used as confirmation of an e-scooter rental. In 16 cases the keywords were not found, and we contacted the clinical provider caring for the patient to clarify whether the scooter in question was a standing e-scooter rental. Three cases were excluded as a result of this follow-up. Charts were also excluded if the above criteria were not met, if the chart reflected a subsequent encounter, or the scooter was determined to be an alternative means of transportation (Figure 1). We could not use an International Classification of Diseases, 10^th^ Revision (ICD-10) code to identify cases as during the study period one did not exist for e-scooter related injury.

**Population Health Research Capsule**

What do we already know about this issue?

*Previous case series examined trends in e-scooter usage and injury patterns related to their use. showing wide variety in the severity and breadth of injuries.*

What was the research question?

*We aimed to provide e-scooter rental injury-incidence rates and characteristics based on ridership data from the scooter companies.*

What was the major finding of the study?

*The incidence of e-scooter rental injuries treated in the emergency department was 2.1 per 10,000 trips.*

How does this improve population health?

*More accurate injury incidence rates from e-scooter rentals will aid future work in understanding injury patterns in comparison to other methods of transport.*

For the prospective arm of the study, research assistants (RA) were notified by an automatic trigger for the words “scooter,” “lime,” or “bird” in the nurse triage note. The care team (attending or resident) also contacted them directly if the e-scooter-related mechanism was ascertained after triage. The RAs were available to respond 24 hours a day/7 days a week. They obtained consent to administer a questionnaire and review the patient’s medical record. The questionnaire included similar chart review elements (age, gender, time of injury, helmet use, intoxication) with the goal of ascertaining more accurate and complete information. The questionnaire also included more specific questions regarding the injury (cause of crash, prior rental use, duration, purpose of rental). Cause of crash was divided into mechanical problem (such as brake problems or loose handlebar); obstacles (curbs, pedestrians, etc); operator error (turned too quickly, multiple riders); street conditions (icy, potholes, etc); struck by vehicle; or unknown. We defined prior rental use as first-time user; intermittent user (five trips or less, but not the first time, or responses of “several times”; and frequent user (over five trips, or responses such as weekly, daily, often). The prospective sample comprised only a quarter of the total patient population (56 of 221).

Reviewers and abstractors were not blinded. For the retrospective arm, charts were abstracted by three physicians at each hospital (AS, VL, CW at BJH and SL, CO, HZ at SLUH) after training on chart review methodology. Senior researchers audited the first 10 charts completed by each abstractor and 10% of subsequent charts. An Excel spreadsheet (Microsoft Corporation, Redmond, WA) and codebook defining variables and preferred chart locations for abstraction of data were generated and shared between institutions. We collected the following variables: age; race; ethnicity; gender; day of week; time of day; drug or alcohol use; helmet use; injury diagnosis; and patient disposition (operating room, admit, home). For the prospective arm one of the senior researchers (LL) trained the RAs on the type of scooter required for inclusion into the study and how to elicit this information. We gave all RAs written survey questions to ask the subjects once they consented to participate in the study. The self-reported responses were recorded in real time. The RAs were given follow-up training, as needed, based on review of their questionnaire data.

We reported frequency statistics of basic demographics (race defined as White, Black or other/unknown and ethnicity defined as non-Hispanic, Hispanic or unknown) as well as intoxication (alcohol or drug use either from lab values, patient reported, or clinically determined), helmet use, disposition, operative repair, and temporal markers (time of day, day of week, month). Time of day was divided into three eight-hour periods – 5 am–12:59 pm, 1 pm–8:59 pm, and 9 pm–4:59 am – to distinguish higher commuter times (morning and afternoon) from more recreational, nighttime usage. When time of injury was not specifically noted either prospectively, in the chart, or through ambulance run sheets, we used time at presentation to the ED as a proxy.

We divided injury type into categories: head injury (diagnosis of concussion, intracranial bleeding of any kind, or traumatic head injury); upper extremity fracture/dislocation; facial fracture; spinal injury (diagnosis of fracture or cord compression); minimal injury (abrasions, lacerations, sprains, strains); and other. Severity scales were measured with Emergency Severity Index (ESI) triage scoring. Patients were considered to have sustained a severe injury if they were triaged as immediate (ESI level 1) or emergent (ESI level 2) or had injuries significant enough to require hospital admission. We used the χ^2^ test to assess for differences between categorical variables in the 2018 and 2019 data to investigate changing trends with the second season of scooter rental. We used Fisher’s exact test to determine differences in groups with more than two variables. We did not make corrections for multiple comparisons.

The two companies that offered e-scooter rentals in our locale during the study period provided aggregate data regarding e-scooter rental usage in the St. Louis metro area. They provided ridership data by hour of day, day of week, and month of year, median distance per ride, average time per ride, and average vehicular speed. The companies did not provide any financial support for the project and were not involved in the analysis or submission. Due to non-disclosure agreements, we were unable to report total numbers of riders, trips, or cumulative mileage.

We used Pearson correlation to determine whether injury rates were correlated with monthly ridership trends and number of injuries. We performed multivariate linear regression to assess for time of day as a potential independent risk factor for an e-scooter-related injury. We entered the following variables via single block entry into the model: average rides per hour and time of day in the three previously listed categories.

## RESULTS

During the 17 months of study, we identified 221 patients with scooter-related ED visits, 94 in the last five months of 2018 and 127 in all of 2019. Basic demographic and injury features of the 221 patients can be found in Table 1.

**Figure 2:**
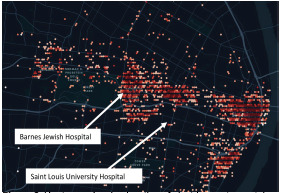
Heat map showing location of electric scooter-rental rides in St. Louis. Darker colors represent a higher density of rides in that location.

The median age of our population was 31 years, 58.8% were male, and 53.8% were White. There were no major differences in age, gender, need for operative repair, triage score, or temporal characteristics between 2018 and 2019. However, more patients were White and non-Hispanic in 2019. Heat map data, a visual representation of the location and density of rides provided by the companies, is shown in Figure 2. We have also indicated the location of the two study centers showing that these facilities are the two hospitals located within the areas with nearly all rental scooter use. No other Level 1 trauma centers are located on the map.

Across the entirety of the time period, the majority of injuries were minor (77.8%) with 90.1% of patients sent home from the ED (discharged, left without being seen, or left against medical advice). There were no deaths. Twenty-two patients (10.0%) were admitted to the hospital, and three (1.4%) of these patients were admitted to the intensive care unit (ICU) or observation unit for closer monitoring. Of the 21 patients diagnosed with head injury, five were found to have an intracranial bleed. Of the 92 patients diagnosed with fracture, 13 were admitted on their index visit for operative repair and another 22 were scheduled or recommended for operative repair as outpatients. Overall, 38% of patients with fractures had or were recommended to have surgery.

**Figure 3:**
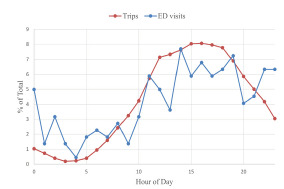
Hourly rides and emergency department visits for electric scooter rental-related injuries by percentage of total. *ED*, emergency department.

Of the 105 patients for whom helmet use was documented, only 4 (3.8%) reported wearing helmets. There was no evidence of association between helmet use and severity of injury. The presence or absence of alcohol or drugs was documented in 108 of the 221 charts reviewed. Fifty-four patients (24.4% of the entire population) were intoxicated with alcohol or drugs. A higher proportion of intoxicated patients met our criteria for severe injury compared to non-intoxicated patients (35.2% vs 16.7%) (Appendix A). In addition, more patients were intoxicated in the overnight period (42.9% from 9 pm – 4:59 am vs 7.5% from 5 am–12:59 pm and 21.9% from 1 pm –8:59 pm) (Appendix B). The highest rate of ED visits and scooter usage was during the afternoon period (Figure 3) with 47.5% of all e-scooter-related ED visits between 1pm–8:59 pm.

Visits to the ED were also more common on the weekends (39.8%) and during September 2018 (14.5%), the second month of rental scooter operation in St. Louis. Severe injuries were more likely to occur in the afternoon (28.6% of severe injuries) compared to the other two time periods (20.6% from 9 pm–4:59 am, and 11.3% from 5 am–12:59 pm) (Appendix C). There was no association between injury severity and month of the year or day of the week.

**Figure 4:**
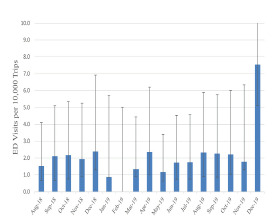
Emergency department visits for electric-scooter rental-related injuries by company-reported trips by month. *Confidence intervals estimated assuming a Poisson distribution. *ED*, emergency department.

The overall incidence of e-scooter rental-related injuries treated in the ED was 2.1 per 10,000 trips (Figure 4) and 2.2 per 10,000 miles.

The questionnaire responses with more specific injury details for the 56 patients from the prospective arm of the study are shown in Table 2.

The data show that the majority (75%) of patients with e-scooter rental-related injuries were not first-time users with 39.3% being frequent users. There was no clear difference between severity or type of injury between first-time users and more experienced users. However, 42.8% of first-time users required operative repair for their injuries compared to only 13.5% of more experienced riders. Most riders were using the scooters for transportation (55.4%). In addition, the cause of injury in most cases was due to road surface conditions (53.2%) or obstacles (14.5%), with few cases involving motor vehicles (10.7%). Most users reported ride length <30 minutes (67.7%) with most frequently a duration of 5 minutes or less (40.3%), which is consistent with company ridership data showing a median trip length of 8.43 minutes/ride.

**Figure 5:**
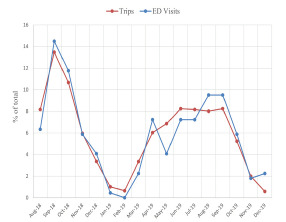
Monthly rides and emergency department visits for electric-scooter rental-related injuries by percentage of total. *ED*, emergency department.

Unique rider data was only available for 2018. Based on this data, each rider took an average of 2.7 trips, with an incidence of 5.7 ED visits per 10,000 unique riders. The average speed was 4.39 miles per hour. Figure 5 shows the general trends of ridership and injuries over the entire study period. Due to specific non-disclosure agreements with both e-scooter companies, the trips taken and ED visits for e-scooter rentals per month are shown using percentages of the total.

The overall number of ED visits and trips per month decreased between 2018 and 2019. However, the number of ED visits by month were very closely correlated with the number of rides per month, with a Pearson correlation coefficient of 0.95. When assessed by time of day using a multivariate linear regression model, ED visits were closely correlated with the number of rides (*P* <0.001). However, riding between the hours of 9 pm and 4:59 am was an independent risk factor for ED visits, after accounting for the number of rides during that time frame (*P* = 0.04) (Appendix D).

## DISCUSSION

Electric scooter rentals have become a popular alternate source of transportation in many cities, both in the US and elsewhere. The media and early reports regarding e-scooter related injuries tended to focus on severe injuries resulting from e-scooter usage. To our knowledge no previously published studies have examined injury rates based on ridership data to better analyze the relative safety of e-scooter use. In St. Louis we found approximately 2.1 visits to the ED per 10,000 rides. Non-peer reviewed data calculated by the Austin, TX, public health department found similar incidence rates (20 per 100,000 trips) over their initial three-month rollout period.^12^

Overall injury rates for e-scooters should be put into context. Comparative data with other forms of non-automobile transportation is difficult to find. The National Highway and Traffic Safety Association reported between 45,000–50,000 bicycle-related injuries nationwide per year in 2014 and 2015^13^; however, this is thought to be a gross underestimate as hospital-based injury rates appear to be about 10 times the injury rate associated with police reports.^14^ The NEISS reports an estimated 123 bicycle-related injuries per 100,000 population.^15^ Based on St. Louis city census population data from 2019, our data show a rate of 51 e-scooter related ED visits per 100,000 population.^16^ Based on the US Centers for Disease Control and Prevention unadjusted injury data from 2017 in combination with estimated national ridership data, there were approximately 8.3 nonfatal injuries and 0.04 fatal, bicycle-related injuries treated in US EDs per 10,000 riders in the adult US population.^17^,^18^ In our data there were approximately 5.7 nonfatal injuries per 10,000 unique riders. Future work comparing local rates of bicycle injury would better quantify this relationship.

The number of ED visits overall was very closely correlated to the number of rides both by hour of day and month of year. This correlation argues that the most significant predictor for the number of e-scooter rental-related ED visits appears to be the number of rides. However, based on the multivariate analysis, riding during nighttime hours (9 pm – 4:59 am) was an independent risk factor. While nighttime injuries were less severe, this time period is associated with an increased injury risk serious enough to require an ED visit. This increased risk may be due to more reckless behaviors or intoxication: 42.9% of patients presenting in the nighttime hours were intoxicated compared to an average of 15% during the other time periods, although decreased visibility and other factors may also have played a role. One company in St. Louis already restricts rides from 2 am – 5 am. Our data would suggest that later nighttime hours present an increased risk of injury for operators of e-scooters. Restrictions on nighttime hour availability should be considered by all vendors.

While research behind incidence trends was previously lacking, our study results focusing on injury patterns are similar to those of previously published studies. An analysis of mass media coverage of e-scooter injuries found 169 incidents with more than 50% involving fatalities or severe injuries.^19^ During the first year of rental scooter usage in the St. Louis metro area we found no fatalities. Among those riders who sustained a scooter-related injury requiring an ED visit, 22.2% met our criteria for a severe injury, 9.5% sustained a head injury, 10% required admission, and 15.8% required operative repair for a fracture.

Case series investigating the number and types of injuries secondary to e-scooter use found varying rates of severe injuries depending on inclusion criteria. Head injuries were the most reported injury, affecting as many as 27.9–40.2% of patients in prior series.^3^,^5^ Our series had a lower incidence of head injuries at 9.5%. This discrepancy may partially be due to differences in reporting. Lacerations and contusions on the scalp were categorized as minor injuries rather than head injuries in this study. Other studies isolating minor from major head trauma have found rates of major head trauma similar to those in this study.^4^,^7^ However, unique vehicular characteristics may also play a role. Among our ridership population the mean speed was under five miles per hour, and few of the patients in the prospective portion of our study had injuries that involved cars (10.7%). Larger cities, with more congested traffic patterns where more riders share the road with cars, may have different results.

Fractures accounted for 30.1–36% of injuries in prior series and 42% in our study. Trivedi et al. reported a 6% admission rate with less than 1% of patients requiring ICU-level care, while Badeau et al. reported a 16% admission rate.^3^,^4^ We found an admission rate of 10% with 1.4% requiring ICU-level care. This is consistent with those studies with similar inclusion criteria, ie, all patients presenting to an ED rather than only trauma activations or patients in trauma registries. In the series published by Dhillon et al., which included only patients requiring services of the trauma team, the ICU admission rate was 20.7%. The Bauer et al. series similarly only included those patients requiring higher level trauma care and reported an admission rate of 43% with 25% requiring ICU-level care.^5^,^6^

This report is the first to assess trends in incidence of e-scooter rental-related ED visits based on reliable ridership data and to compare the incidence longitudinally over time, seasonally by month, and by time of day. Further evaluation of incidence trends is important to help guide necessary safety choices.

## LIMITATIONS

There are several limitations we must note. Although the data come from the only two trauma centers located within the city limits and corresponds to the areas of most frequent scooter usage, it is likely that some patients with scooter-related injuries were seen elsewhere. Several patients in the data set were transferred after initially being seen at urgent care centers. Whereas less severely injured riders may not have presented to our facilities, we believe that the most seriously injured riders were ultimately seen in one of the two downtown EDs, either through initial transport or by transfer. While our data provide one of the longest study periods for e-scooter rental surveillance, it was still isolated to 17 months. We believe this is likely to capture the majority of clear trends in injuries based on initial rollout and novelty; however, there is still a possibility that these trends could manifest with further long-term study.

Additionally, there is no ICD-10 code specific for injuries related to e-scooters, and the search strategy may have missed cases. We also did not include any pediatric cases. While the companies restrict users under 18, there are still likely pediatric cases missed by this study. It is also possible that in the retrospective portion of the study we could have included injuries to individuals who personally owned electric scooters. However, we consider it to be unlikely or a minimal contribution as we used every measure possible to exclude injuries that occurred with personally owned e-scooters (searching for company names or “rental,” discussion with providers), and none of the patients prospectively screened for inclusion in the study were riding a personally owned e-scooter. We also have limitations in the data, since many of the questions such as helmet use, intoxication, and causation were not consistently documented.

## CONCLUSION

The number of electric scooter rental-related injuries seen in our two adult Level I ED trauma centers in the St. Louis metro area was relatively low, correlated closely with overall number of rides, and decreased in both quantity and severity between the first and second years of operation. Documented helmet use was rare. Importantly, the injuries that did occur were occasionally serious, including a small number of intracranial injuries and a large percentage of fractures requiring operative repair.

## Supplementary Information



## Figures and Tables

**Table 1:** Demographic and characteristics related to patients with scooter-related injuries who presented to the emergency department.

	2018 Number (%)	2019 Number (%)	Total Number (%)
Demographic characteristics			
Age			
18–25	31 (33%)	36 (28.3%)	67 (30.3%)
26–40	36 (38.3%)	54 (42.5%)	90 (40.7%)
41–64	26 (27.7%)	37 (29.1%)	63 (28.5%)
≥65	1 (1.1%)	0 (0%)	1 (0.5%)
Age (Median [IQR])	31 (21.5–40.5)	31 (24–42)	31 (22–40)
Gender			
Male	52 (55.3%)	78 (61.4%)	130 (58.8%)
Female	42 (44.7%)	49 (38.6%)	91 (41.2%)
Race*			
White	43 (45.7%)	76 (59.8%)	119 (53.8%)
Black	35 (37.2%)	47 (37.0%)	82 (37.1%)
Other/Unknown	16 (17%)	4 (3.1%)	20 (9.0%)
Ethnicity*			
Non-Hispanic	85 (90.4%)	123 (96.9%)	208 (94.1%)
Hispanic	2 (2.1%)	3 (2.4%)	5 (2.3%)
Unknown	7 (7.4%)	1 (0.8%)	8 (3.6%)
Injury characteristics			
Intoxication*			
Yes	17 (18.1%)	37 (29.1%)	54 (24.4%)
No	10 (10.6%)	44 (34.6%)	54 (24.4%)
Unknown	67 (71.3%)	46 (36.2%)	113 (51.1%)
Helmet*			
Yes	1 (1.1%)	3 (2.4%)	4 (1.8%)
No	31 (33%)	70 (55.1%)	101 (45.7%)
Unknown	62 (66%)	54 (42.5%)	116 (52.5%)
Main injury*			
Head injury	12 (12.8%)	9 (7.1%)	21 (9.5%)
Lower extremity fracture	10 (10.6%)	14 (11.0%)	24 (10.9%)
Upper extremity fracture	13 (13.8%)	35 (27.6%)	48 (21.7%)
Facial fracture	4 (4.3%)	14 (11.0%)	18 (8.1%)
Spinal injury	2 (2.1%)	1 (0.8%)	3 (1.4%)
Minor injury	48 (51.1%)	49 (38.6%)	97 (43.9%)
Other	5 (5.3%)	5 (3.9%)	10 (4.5%)
Disposition*			
Floor	10 (10.6%)	9 (7.1%)	19 (8.6%)
ICU or OU	3 (3.2%)	0 (0%)	3 (1.4%)
Discharge	76 (80.9%)	114 (89.8%)	190 (86%)
Left without being seen	5 (5.3%)	2 (1.6%)	7 (3.2%)
Left against medical advice	0 (0%)	2 (1.6%)	2 (0.9%)
Surgical repair	14 (14.9%)	21 (16.5%)	35 (15.8%)
ESI triage			
1 (immediate)	1 (1.1%)	0 (0%)	1 (0.5%)
2 (emergent)	20 (21.3%)	17 (13.4%)	37 (16.7%)
3 (urgent)	55 (58.5%)	85 (66.9%)	140 (63.3%)
4 (less urgent)	17 (18.1%)	24 (18.9%)	41 (18.6%)
5 (least urgent)	1 (1.1%)	0 (0%)	1 (0.5%)
Unknown	0 (0%)	1 (0.8%)	1 (0.5%)
Severe injuryǂ	29 (30.9%)	20 (15.7%)	49 (22.2%)
Temporal characteristics			
Day of week			
Sunday	20 (21.3%)	20 (15.7%)	40 (18.1%)
Monday	10 (10.6%)	14 (11%)	24 (10.9%)
Tuesday	12 (12.8%)	16 (12.6%)	28 (12.7%)
Wednesday	12 (12.8%)	15 (11.8%)	27 (12.2%)
Thursday	9 (9.6%)	19 (15.0%)	28 (12.7%)
Friday	10 (10.6%)	16 (12.6%)	26 (11.8%)
Saturday	21 (22.3%)	27 (21.3%)	48 (21.7%)
Time of day			
5 AM–12:59 PM	26 (27.7%)	27 (21.3%)	53 (24%)
1 PM–8:59 PM	45 (47.9%)	60 (47.2%)	105 (47.5%)
9 PM–4:59 AM	23 (24.5%)	40 (31.5%)	63 (28.5%)

* Fisher’s exact test < 0.05.

*IQR*, interquartile range; *ICU*, intensive care unit; *OU*, observation unit.

ǂ χ^2^ < 0.05.

*ESI*, Emergency Severity Index.

**Table 2:** Prospective data questionnaire responses.

Questionnaire responses	Number (%)
Injury trigger	
Mechanical error	2 (3.2%)
Obstacles	9 (14.5%)
Operator error	4 (6.5%)
Road surface conditions	33 (53.2%)
Struck by vehicle	6 (10.7%)
Unknown	2 (3.6%)
User characteristics	
First-time user	14 (25%)
Intermittent user	20 (35.7%)
Frequent user	22 (39.3%)
Purpose	
Transportation to/from work/school	14 (25%)
Other transportation	17 (30.4%)
Recreational	24 (42.9%)
Unknown	1 (1.8%)
Duration	
≤ 5 min	25 (40.3%)
>5 min and ≤ 30 min	17 (27.4%)
>30 min	12 (19.4%)
Unknown	2 (3.2%)

*Min*, minutes.
