# Supplementary material for: Incidence of Emergency Department Visits for Electric Rental Scooters Using Detailed Ridership Data
Source: West J Emerg Med. 2022 Jan 31;23(2):174–82. doi: 10.5811/westjem.2021.6.51101 (PMC8967461; doi:10.5811/westjem.2021.6.51101)
Supplement: Supplementary file 1 [file wjem-23-174-s001.docx]

Appendix A: ED visits by intoxication and severity

|  | Intoxicated | Not intoxicated | Unknown |
| --- | --- | --- | --- |
| Non-severe | 35 (64.8%) | 45 (83.3%) | 92 (81.4%) |
| Severe | 19 (35.2%) | 9 (16.7%) | 21 (18.5%) |

Fischer's Exact Test = .036

Appendix B: ED visits by intoxication and time period

|  | 5AM-12:59PM | 1PM-8:59PM | 9PM-4:59AM |
| --- | --- | --- | --- |
| Intoxicated | 4 (7.5%) | 23 (21.9%) | 27 (42.9%) |
| Not intoxicated | 17 (32.1%) | 29 (27.6%) | 8 (12.7%) |
| Unknown | 32 (60.4%) | 53 (50.5%) | 28 (44.4%) |

Fischer's Exact Test < .001

Appendix C: ED visits by time period and severity

|  | 5AM-12:59PM | 1PM-8:59PM | 9PM-4:59AM |
| --- | --- | --- | --- |
| Non-Severe | 47 (88.7%) | 75 (71.4%) | 50 (79.3%) |
| Severe | 6 (11.3%) | 30 (28.6%) | 13 (20.6%) |

Fischer's Exact Test = .043

Appendix D: Multivariate Analysis

|  | Unstandardized Coefficients | |  | | | | |
| --- | --- | --- | --- | --- | --- | --- | --- |
|  | B | Std Error | Standardized Coeffecients Beta | t | sig | lower bound | upper bound |
| Constant | 1.592 | 1.55 |  | 1.027 | 0.317 | -1.641 | 4.824 |
| Average trips per hour | 0.025 | 0.006 | 0.97 | 4.406 | <.001 | 0.013 | 0.037 |
| Time category 1-8:59PM | -0.134 | 2.112 | -0.013 | -0.063 | 0.95 | -4.538 | 4.271 |
| Time category 9PM-4:59AM | 3.382 | 1.558 | 0.337 | 2.171 | 0.042 | 0.133 | 6.632 |

| R | R^2^ | Adjusted R^2^ | Std error of estimate |
| --- | --- | --- | --- |
| 0.82 | 0.673 | 0.624 | 2.961 |
